# Dynamic Growth and Shrinkage Govern the pH Dependence of RecA Filament Stability

**DOI:** 10.1371/journal.pone.0115611

**Published:** 2015-01-21

**Authors:** Sung Hyun Kim, Jeehae Park, Chirlmin Joo, Doseok Kim, Taekjip Ha

**Affiliations:** 1 Department of Physics and Interdisciplinary Program of Integrated Biotechnology, Sogang University, Seoul, Korea; 2 Center for Biophysics and Computational Biology, University of Illinois at Urbana-Champaign, Urbana, Illinois, United States of America; 3 Kavli Institute of NanoScience, Department of BioNanoScience, Delft University of Technology, Delft, The Netherlands; 4 Howard Hughes Medical Institute, Department of Physics, University of Illinois at Urbana-Champaign, Urbana, Illinois, United States of America; Tulane University Health Sciences Center, UNITED STATES

## Abstract

RecA proteins form a long stable filament on a single-stranded DNA and catalyze strand exchange reaction. The stability of RecA filament changes dramatically with pH, yet its detailed mechanism is not known. Here, using a single molecule assay, we determined the binding and dissociation rates of RecA monomers at the filament ends at various pH. The pH-induced rate changes were moderate but occurred in opposite directions for binding and dissociation, resulting in a substantial increase in filament stability in lower pH. The highly charged residues in C-terminal domain do not contribute to the pH dependent stability. The stability enhancement of RecA filament in low pH may help the cell to cope with acidic stress by fine-tuning of the binding and dissociation rates without losing the highly dynamic nature of the filament required for strand exchange.

## Introduction

RecA protein is essential for repairing damaged chromosomal DNA by mediating homologous recombination [1–3]. There is a collection of evidence that points to the role of RecA in dealing with acidic stress. Deletion of RecA in *E. coli* was found to be lethal when subjected to an acidic environment [4]. Acid tolerance is vital to *E. coli* as it needs to pass through the acidic stomach of vertebrate. Similarly, RecA was found to be up-regulated in *S. mutans*, when grown in acidic condition [5]. Therefore, enhanced filament formation and strand exchange activity under low pH conditions would help the host cell to survive. Yet, the details of how the stability of RecA filament is regulated by pH remain unclear.

RecA proteins form a filament of helical structure on a single stranded (ss) DNA [6,7]. Filament formation is initiated by binding (nucleation) of five to six monomers simultaneously on a ssDNA [8,9]. The nucleated short filament then grows by addition of monomers at either end, covering as many as thousands of nucleotides in the ssDNA. Monomer dissociation also takes place at the filament ends, which puts the filament ends in dynamic equilibrium between monomer binding and dissociation. The difference between binding and dissociation rates determines whether the filament grows or shrinks. In our previous report [9], we have shown that the binding rate at the 3’ end is much higher than the dissociation rate at physiological concentration of RecA, whereas the binding and the dissociation rates are comparable at the 5’ end. Therefore, the kinetics at 5’ end is crucial for determining the overall stability of the RecA filament.

Stable filament formation [10] and enhanced strand exchange reaction [11–13] at low pH have been well-documented. It was proposed that suppression of RecA dissociation is responsible for the stable filament formation at low pH while the binding is not altered [14]. However, it is puzzling why the dissociation process is affected by pH given the fact that the monomer dissociation is strongly coupled with ATP hydrolysis that is pH-independent [11,15,16]. Furthermore, a recent single molecule study reported enhancement in both initial nucleation and consequent growth rates of a filament at low pH, suggesting that pH may affect both binding and dissociation processes [17]. Also, pH-dependent enhancement of nucleation of RecA proteins on a dsDNA was observed in another single-molecule manipulation study [18], yet the details of the RecA dynamics at the filament ends at high resolution are still missing.

In this study, using the single-molecule fluorescence resonance energy transfer (smFRET) technique [19,20], we directly observed monomer binding and dissociation events at the 5’ end to elucidate the details of the molecular mechanism of the pH-dependent RecA filament stability.

## Materials and Methods

### Reaction Condition

RecA was purchased from *New England Biolabs* and used without further purification. Reaction was performed with a solution consisting of 10 mM Mg(OAc)_2_, 100 mM NaOAc, and 10 mM Tris-OAc at pH 7.5 with 1 mM ATP (Sigma) unless otherwise specified. Instead of Tris buffer, the same concentrations of Mes and Mops were used to maintain the pH in the range from 6.0 to 6.3, and from 6.6 to 7.0, respectively. All the measurements were carried out in room temperature.

### DNA

Cy5- and Biotin-labeled single strand (ss) DNA with the sequence of 5′-Cy5-GCC TCG CTG CCG TCG CCA –biotin-3′ was purchased from IDTDNA. Cy3 dye were labeled through a amine modified thymine on the ssDNA of which sequence is 5′-TGG CGA CGG CAG CGA GGC-(T)_10_-T*-(T)_49_-3’, where T* denotes the cy3 labeling position. The two ssDNAs were hybridized to a partial duplex DNA with ss-tail.

### Single-molecule FRET Assay

Experimental details on single-molecule FRET assay are described in the previous work [9]. Fluorescently labeled DNA was immobilized on polyethyleneglycol (PEG) coated quartz slide surface to minimize non-specific adsorption of protein to the surface. At the buffer pH lower than 7.5, we further treated PEG surface with disuccinimidyl tartarate (PIERCE) to eliminate free amine groups on the PEG surface that can electrostatically attract the phosphate backbone of DNA. Fluorescence signal after illumination with 532 nm laser (CrystaLaser) via total internal reflection was collected with an objective lens (Olympus, NA 1.2 water immersion) and spectrally separated with a dichroic mirror before being imaged on an electron-multiplying charge-coupled device (EMCCD). Fluorescence intensity time traces were extracted out from a movie taken and FRET efficiency was calculated with home-built software. Most likely FRET time trajectories were estimated with hidden Markov method [21]. FRET histograms were built from thousands of single molecules of which FRET values were determined by averaging the first 10 data points from the traces.

## Results and Discussion

**Figure 1:**
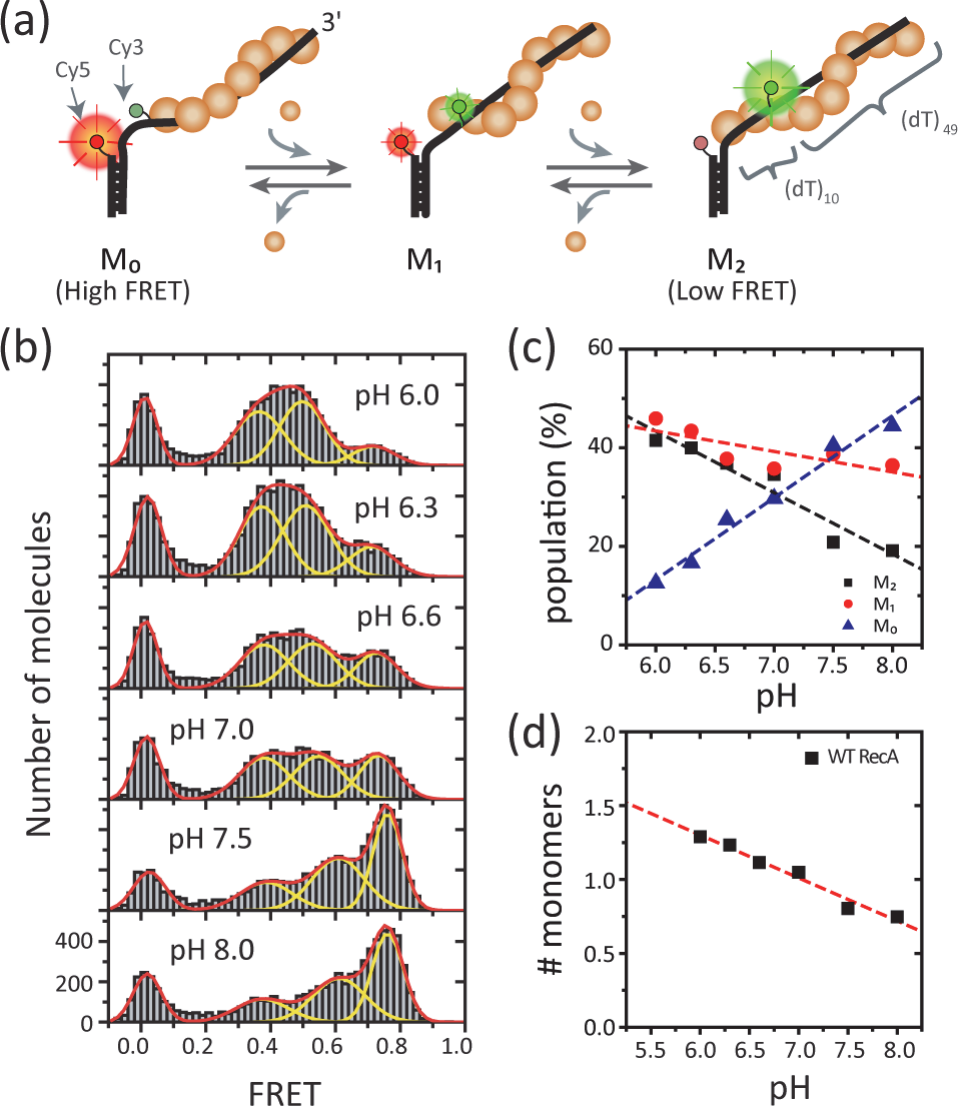
pH dependent stability change of RecA filament at 5′-disassembly end. (a) A model for RecA monomer binding and dissociation at the filament end. (b) Single molecule FRET histograms at different pH. The histogram was fit with three Gaussian peaks for M_0_, M_1_, and M_2_ states, respectively, with additional peak at 0 for donor-only molecules. (c) The populations for each peak found in (b) at various pHs (symbols). Dashed lines are eye guides. (d) Average number of monomers bound between donor and acceptors (filled rectangles) calculated from (c): N_average_ = 2*P(M_2_) + 1*P(M_1_) +0*P(M_0_), where P(M_i_) is the population in M_i_ state. Red dashed line is a linear fit to the data with a slope −0.29.

To observe binding and dissociation events from a RecA filament at the single monomer level, we designed a partial duplex DNA with a 60 nt long 3’ single-stranded overhang. We placed the donor (Cy3) and acceptor (Cy5) at the junction of the double and single strand region with 10-nt separation, such that only a few RecA monomers may bind between them (**Fig. 1A**). Because the fluorophores were located at the 5’ side of the ss-overhang and RecA filament formation is possible only on ssDNA in our buffer condition (i.e. the filament cannot continue onto the double stranded region), FRET fluctuations here would monitor the dynamics at the 5’ end of RecA filament [9,18]. We immobilized the partial duplex DNA on a polymer-passivated quartz slide glass and measured fluorescence from individual molecules by using a total internal reflection fluorescence microscopy. We obtained single molecule FRET histograms in the presence of RecA (1 μM) at various pHs ranging from 6.0 to 8.0 (**Fig. 1B**) and determined how stably RecA was bound at the 5′-disassembly end. In the histograms, three distinct peaks, located at 0.4, 0.6 and 0.75 were observed. An extra peak located at 0 is from the donor-only species and excluded from analysis. The highest FRET peak corresponds to the naked ssDNA without bound RecA monomers between the probes and designated as M_0_ state. Binding of an additional monomer causes a decrease in FRET efficiency as the ssDNA region is stretched by the filament growth. Thus, we assigned the intermediate FRET populations at 0.6 and 0.4 as M_1_ (one-monomer bound state) and M_2_ (two-monomers bound state), respectively. To ensure that the observations are not due to a possible artifact caused by direct influence of pH on the photophysical properties of the fluorophores, we have measured smFRET of RecA filaments formed with a non-hydrolysable ATP analogue, ATPγS, so that the filament remains stable without any dissociation (S1A **Fig**.). The FRET values obtained from the ATPγS/RecA filaments showed no differences when subjected to pH of 6.0, 7.0 or 8.0 (S1B **Fig**.). Also, we did not observe any FRET changes in single molecule time traces measured at different pH, indicating that any photophysical effects of pH are minimal (S1C-E **Fig**.).

Consistent with the previous reports of the enhanced filament stability at low pH [10–14,17], single molecule populations were biased towards low FRET (M_2_) at low pH and towards high FRET (M_0_) at high pH (**Fig. 1B and 1C**). As a measure of filament stability, we calculated the average number of RecA monomers bound between the donor and acceptor (**Fig. 1D**). The average number of monomers linearly decreased with increasing pH (the slope from linear fit was −0.29). The linear dependency in the filament growth is consistent with the recent single-molecule result [17]. Interestingly, we observed more than 50% of the populations are still in M_1_ or M_0_ states even at the lowest pH condition (pH 6.0) whereas complete suppression of dissociation was predicted in a previous report [14].

**Figure 2:**
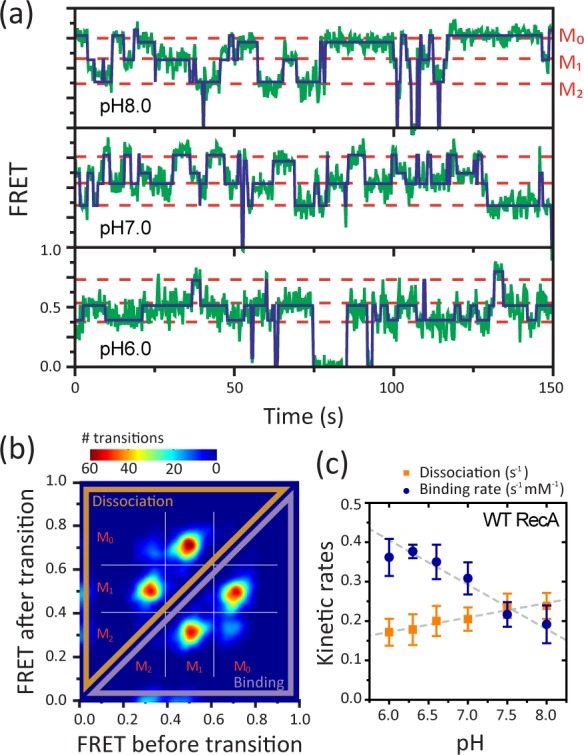
Real-time observation of monomer binding and dissociation events. (a) Representative FRET time traces measured at pH 8.0, pH 7.0, and pH 6.0 (green solid lines) with most likely FRET values assigned by hidden Markov modeling (dark blue solid lines). (b) A representative transition density plot (TDP) measured at pH 7.0. 7,560 transitions were identified by the hidden Markov modeling from 94 molecules. The transitions were classified into four groups: two for monomer binding and two for monomer dissociation. (c) The binding (blue) and dissociation (orange) rates as a function of pH measured with wild type RecA. The error bars are standard deviations from three independent measurements. The slopes obtained by linear fit (dashed lines) to the dissociation rates and the binding rates are 0.05 and −0.10, respectively.

With the successful recapitulation of the pH-dependent filament stability using our single molecule assay, we then carried out real-time measurements of binding and dissociation events to obtain the kinetic rates. Typical FRET time traces obtained at different pHs are shown in **Fig. 2A** (green lines). Monomer binding and dissociation events were identified as sudden jumps in the traces. For a quantitative analysis, we determined the FRET states and transition points with hidden Markov modeling (HMM) analysis (**Fig. 2A**, blue solid lines) [21], which also yields the transition rates between the states. Once all the transitions between the discrete FRET states were identified, we built a two-dimensional histogram (transition density plot, TDP) of the transitions to classify the transitions into groups (**Fig. 2B**). We observed four different groups of the transitions, two of which were the binding events and the other two were the dissociation events. For each group of the transitions, we calculated the average values of the transition rates determined by the HMM analysis (**Fig. 2C**). The dissociation rate showed a moderate decrease as pH was lowered (**Fig. 2C, orange**). The effect of pH on the binding rate was larger and in the opposite direction, increasing as pH was lowered (**Fig. 2C, blue**). Also notable is the observation that the dissociation rate did not approach 0 even at the lowest pH value examined (**Fig. 2C, orange**), showing that dissociation is not suppressed entirely at pH 6.0. The slope of the binding rate vs. pH curve was twice as high as that of the dissociation rate vs. pH, suggesting that pH has a stronger effect on the binding rate than on the dissociation rate.

**Figure 3:**
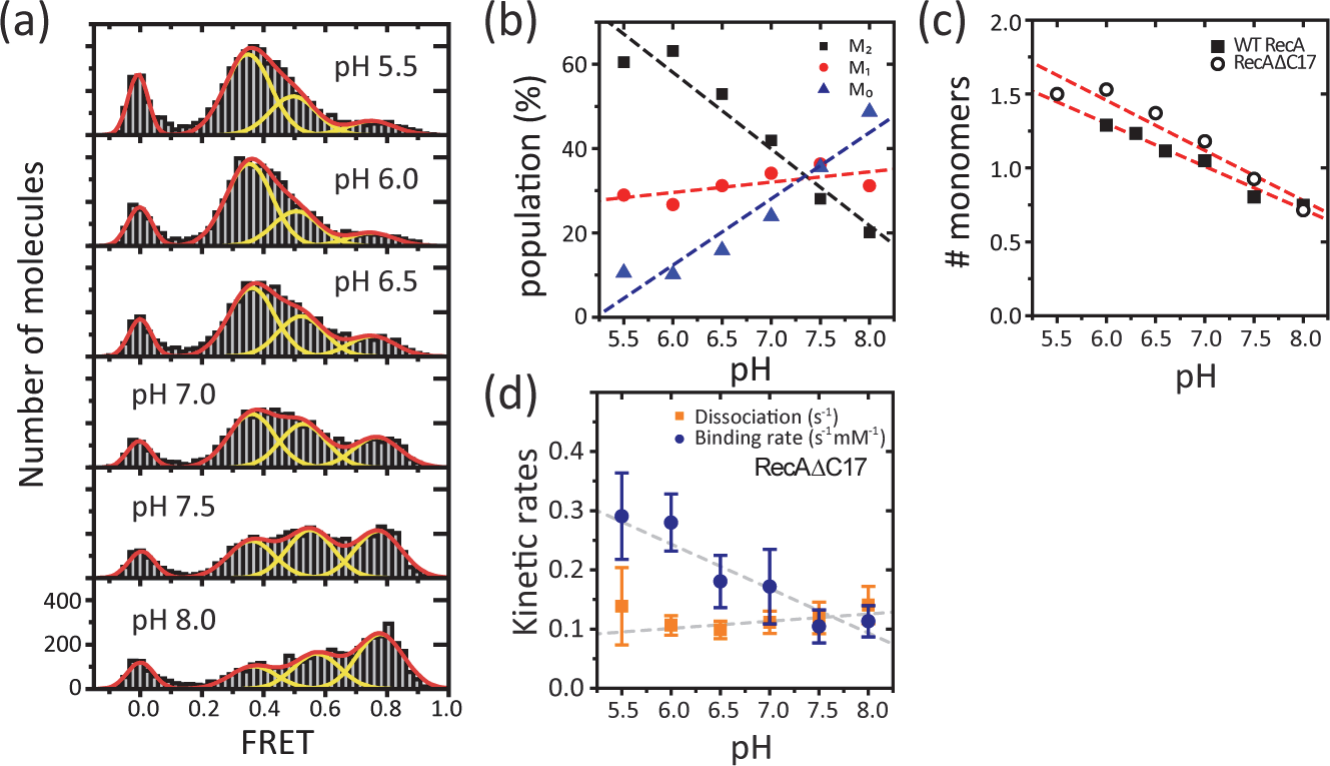
pH Dependence of the RecAΔC17 Filament at the 5′-disassembly End. (a) Single molecule FRET histograms obtained at various pH with a RecA mutant of which negatively charged 17 amino acids in CTD are truncated (RecAΔC17). 0.5 μM RecA ΔC17 and 1 mM ATP were added in the reaction buffer with the same buffer condition as in Fig. 1. The histogram was fit with three Gaussian peaks for M_0_, M_1_, and M_2_ state, respectively, with additional peak at 0 for donor-only molecules. (b) The population for each peaks (symbols) found in (a) at various pH. Dashed lines are eye guides. (c) Average number of monomers bound between donor and acceptors (open circles for RecAΔC17) calculated from (b): N_average_ = 2*P(M_2_) + 1*P(M_1_) +0*P(M_0_), where P(M_i_) is the population in M_i_ state. Red dashed line is a linear fit to the data with a slope −0.34 for RecAΔC17. Data obtained with wild type RecA in Fig. 1D is also shown for comparison (filled rectangles). (d) The binding (blue) and dissociation (orange) rates of RecAΔC17 at different pHs. The error bars are standard deviations from three independent measurements. The slopes obtained by linear fit (dashed lines) are 0.01 and −0.07 for the dissociation and the binding rates, respectively.

The pH of the solution would change the surface charge density of the RecA protein, which would in turn affect the electrostatic interaction between adjacent RecA monomers in a filament. In the crystal structures [7,22], three charged surfaces were found: 1) positively charged N-terminal domain (NTD), 2) negatively charged surface in the core domain, 3) negatively charged C-terminal domain (CTD). Among these three charged surfaces, the interaction between the NTD and the core domain via electrostatic force is considered essential for filament formation because it is the major contact between two adjacent monomers in the filament [23]. In contrast, CTD may not interact with neighboring monomers or substrate DNA [7]. Indeed, consistent with the above picture, pH-dependent filament stability and dynamics for a RecA mutant lacking 17 highly charged amino acids in CTD was indistinguishable from the wild type (**Fig. 3**). Population change in the FRET histograms (**Fig. 3A-C**) and the monomer binding and dissociation rates at different pH’s (**Fig. 3D**) observed with the mutant were similar to that observed with wide type. Thus, the pH dependent kinetics change in monomer binding and dissociation is unlikely to be due to the charged residues in CTD. CTD, instead of affecting filament stability and dynamics through its charged surface, is believed to interact with another filament and the secondary homologous DNA [24] and with other proteins such as SSB [25].

In this study we have utilized single-molecule FRET to investigate the stability of a RecA filament as a function of pH. We show that stable filament formation at low pH is achieved by both increasing the binding rate and moderately decreasing the dissociation rate. Furthermore, we show that the highly charged residues in C-terminal domain are not responsible for the pH dependent stability. Based on our observations, we propose that the electrostatic interaction of the core domain with the NTD from the next monomer in the filament is the major origin of the pH dependence. The stability enhancement of RecA filament in low pH may help the cell to cope with acidic stress and occurs via fine-tuning of the binding and dissociation rates without losing the highly dynamic nature of the filament required for strand exchange.

## Supporting Information

S1 Fig Single molecule FRET histograms and time traces measured from RecA filaments formed with ATPγS reveal no direct influence of pH on Cy3—Cy5 dye pair. (a) A schematic model of RecA filament formed with ATPγS. The dyes are labeled internally with 9 nt separation on the single stranded overhang consisting of polythymine. The distance from the junction to the dye pair is 30 nt and the distance from 3’ end is 22 nt. Double stranded region for surface immobilization is the same as that used in Fig. 1.
(b) Single molecule FRET histograms measured at different pHs. While the bare DNA showed a peak at E∼0.8 (top panel), RecA filaments formed with ATPγS showed a peak at E∼0.4 regardless of pH. Because the dye separation is 9nt, only a stable three monomer binding state between the dyes is expected. A peak at 0 is due to donor-only molecules.
(c-e) Representative single molecule intensity and FRET traces measured at different pH. For each pH condition, two representative traces were shown. Traces in the left panels were chosen to show a single-step photo bleaching event of acceptor at about 35∼40 sec.
(PDF)

## References

[1] Kowalczykowski SC (2000) Initiation of genetic recombination and recombination-dependent replication. Trends Biochem Sci 25: 156–165. 10.1016/S0968-0004(00)01569-3 10754547

[2] Lusetti SL, Cox MM (2002) The bacterial RecA protein and the recombinational DNA repair of stalled replication forks. Annu Rev Biochem 71: 71–100. 10.1146/annurev.biochem.71.083101.133940 12045091

[3] Kowalczykowski SC, Dixon DA, Eggleston AK, Lauder SD, Rehrauer WM (1994) Biochemistry of homologous recombination in Escherichia coli. Microbiol Rev 58: 401–465. 7968921 10.1128/mr.58.3.401-465.1994 PMC372975

[4] Jeong KC, Hung KF, Baumler DJ, Byrd JJ, Kaspar CW (2008) Acid stress damage of DNA is prevented by Dps binding in Escherichia coli O157:H7. BMC Microbiol 8: 181 10.1186/1471-2180-8-181 18922164 PMC2588596

[5] Len AC, Harty DW, Jacques NA (2004) Stress-responsive proteins are upregulated in Streptococcus mutans during acid tolerance. Microbiology 150: 1339–1351. 10.1099/mic.0.27008-0 15133096

[6] Egelman EH, Stasiak A (1993) Electron-Microscopy of Reca-DNA Complexes—2 Different States, Their Functional-Significance and Relation to the Solved Crystal-Structure. Micron 24: 309–324.

[7] Chen Z, Yang H, Pavletich NP (2008) Mechanism of homologous recombination from the RecA-ssDNA/dsDNA structures. Nature 453: 489–484. 10.1038/nature06971 18497818

[8] Galletto R, Amitani I, Baskin RJ, Kowalczykowski SC (2006) Direct observation of individual RecA filaments assembling on single DNA molecules. Nature 443: 875–878. 10.1038/nature05197 16988658

[9] Joo C, McKinney SA, Nakamura M, Rasnik I, Myong S, et al. (2006) Real-time observation of RecA filament dynamics with single monomer resolution. Cell 126: 515–527. 10.1016/j.cell.2006.06.042 16901785

[10] McEntee K, Weinstock GM, Lehman IR (1981) Binding of the recA protein of Escherichia coli to single- and double-stranded DNA. J Biol Chem 256: 8835–8844. 7021553

[11] Muench KA, Bryant FR (1990) An obligatory pH-mediated isomerization on the [Asn-160]recA protein-promoted DNA strand exchange reaction pathway. J Biol Chem 265: 11560–11566. 2142155

[12] Vaze MB, Muniyappa K (1999) RecA protein of Mycobacterium tuberculosis possesses pH-dependent homologous DNA pairing and strand exchange activities: implications for allele exchange in mycobacteria. Biochemistry 38: 3175–3186. 10.1021/bi9819125 10074373

[13] Pinsince JM, Muench KA, Bryant FR, Griffith JD (1993) Two mutant RecA proteins possessing pH-dependent strand exchange activity exhibit pH-dependent presynaptic filament formation. J Mol Biol 233: 59–66. 10.1006/jmbi.1993.1484 8377192

[14] Arenson TA, Tsodikov OV, Cox MM (1999) Quantitative analysis of the kinetics of end-dependent disassembly of RecA filaments from ssDNA. J Mol Biol 288: 391–401. 10.1006/jmbi.1999.2705 10329149

[15] Weinstock GM, McEntee K, Lehman IR (1981) Hydrolysis of nucleoside triphosphates catalyzed by the recA protein of Escherichia coli. Characterization of ATP hydrolysis. J Biol Chem 256: 8829–8834. 7021552

[16] Kim SH, Joo C, Ha T, Kim D (2013) Molecular mechanism of sequence-dependent stability of RecA filament. Nucleic Acids Research 41: 7738–7744. 10.1093/nar/gkt570 23804763 PMC3763553

[17] Bell JC, Plank JL, Dombrowski CC, Kowalczykowski SC (2012) Direct imaging of RecA nucleation and growth on single molecules of SSB-coated ssDNA. Nature 491: 274–278. 10.1038/nature11598 23103864 PMC4112059

[18] Fu H, Le S, Muniyappa K, Yan J (2013) Dynamics and Regulation of RecA Polymerization and De-Polymerization on Double-Stranded DNA. PLoS One 8: e66712 23825559 10.1371/journal.pone.0066712 PMC3688958

[19] Ha T, Enderle T, Ogletree DF, Chemla DS, Selvin PR, et al. (1996) Probing the interaction between two single molecules: fluorescence resonance energy transfer between a single donor and a single acceptor. Proc Natl Acad Sci U S A 93: 6264–6268. 10.1073/pnas.93.13.6264 8692803 PMC39010

[20] Ha T, Kozlov AG, Lohman TM (2012) Single-molecule views of protein movement on single-stranded DNA. Annu Rev Biophys 41: 295–319. 10.1146/annurev-biophys-042910-155351 22404684 PMC3719979

[21] McKinney SA, Joo C, Ha T (2006) Analysis of single-molecule FRET trajectories using hidden Markov modeling. Biophys J 91: 1941–1951. 10.1529/biophysj.106.082487 16766620 PMC1544307

[22] Story RM, Weber IT, Steitz TA (1992) The Structure of the Escherichia-Coli Reca Protein Monomer and Polymer. Nature 355: 318–325. 10.1038/355318a0 1731246

[23] Mikawa T, Masui R, Ogawa T, Ogawa H, Kuramitsu S (1995) N-terminal 33 amino acid residues of Escherichia coli RecA protein contribute to its self-assembly. J Mol Biol 250: 471–483. 10.1006/jmbi.1995.0391 7616568

[24] Lusetti SL, Shaw JJ, Cox MM (2003) Magnesium ion-dependent activation of the RecA protein involves the C terminus. J Biol Chem 278: 16381–16388. 10.1074/jbc.M212916200 12595538

[25] Eggler AL, Lusetti SL, Cox MM (2003) The C terminus of the Escherichia coli RecA protein modulates the DNA binding competition with single-stranded DNA-binding protein. J Biol Chem 278: 16389–16396. 10.1074/jbc.M212920200 12598538

