# Supplementary figures and images for: Dynamic Growth and Shrinkage Govern the pH Dependence of RecA Filament Stability
Source: PLoS One. 2015 Jan 21;10(1):e0115611. doi: 10.1371/journal.pone.0115611 (PMC4301630; doi:10.1371/journal.pone.0115611)

Figure S1

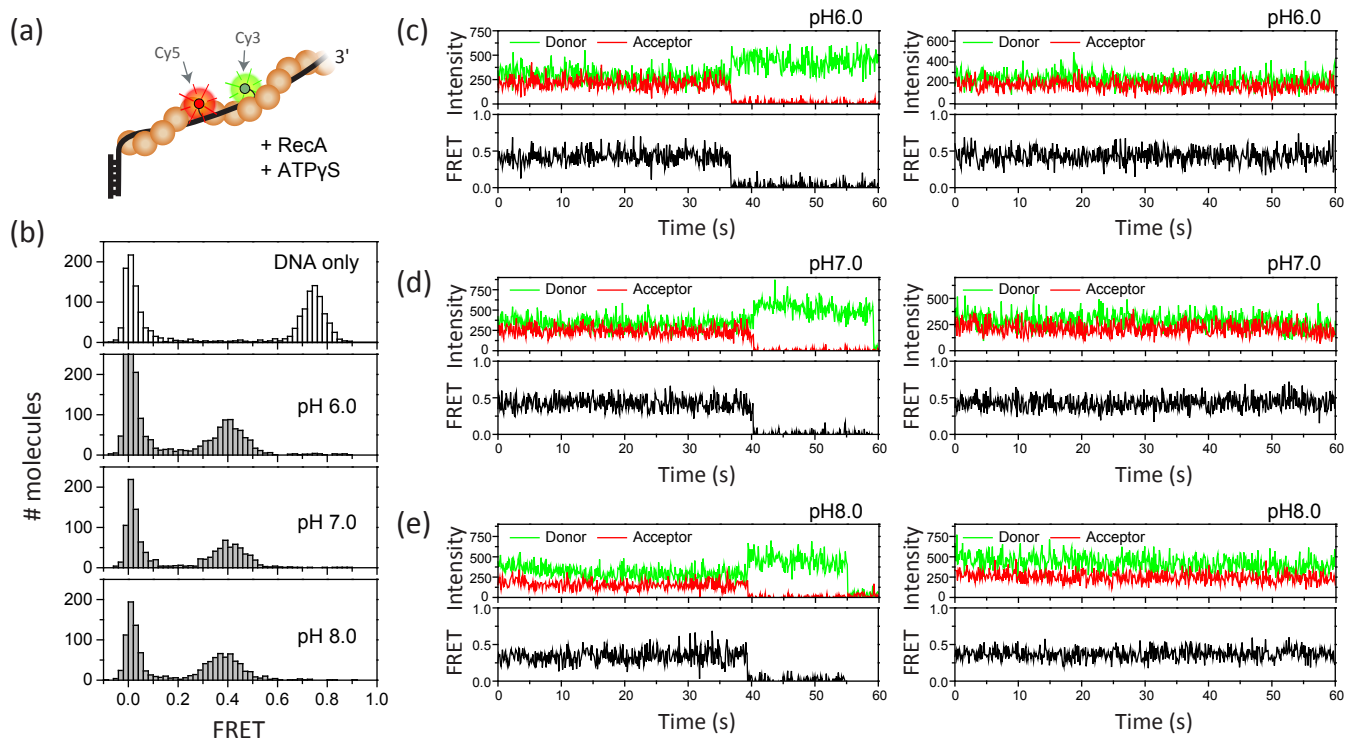

Supplement: S1 Fig — (a) A schematic model of RecA filament formed with ATPγS. The dyes are labeled internally with 9 nt separation on the single stranded overhang consisting of polythymine. The distance from the junction to the dye pair is 30 nt and the distance from 3’ end is 22 nt. Double stranded region for surface immobilization is the same as that used in Fig. 1. (b) Single molecule FRET histograms measured at different pHs. While the bare DNA showed a peak at E∼0.8 (top panel), RecA filaments formed with ATPγS showed a peak at E∼0.4 regardless of pH. Because the dye separation is 9nt, only a stable three monomer binding state between the dyes is expected. A peak at 0 is due to donor-only molecules. (c-e) Representative single molecule intensity and FRET traces measured at different pH. For each pH condition, two representative traces were shown. Traces in the left panels were chosen to show a single-step photo bleaching event of acceptor at about 35∼40 sec. (PDF) [file pone.0115611.s001.pdf]
